# Antioxidant Defense in the Toughest Animals on the Earth: Its Contribution to the Extreme Resistance of Tardigrades

**DOI:** 10.3390/ijms25158393

**Published:** 2024-08-01

**Authors:** Izabela Sadowska-Bartosz, Grzegorz Bartosz

**Affiliations:** Laboratory of Analytical Biochemistry, Institute of Food Technology and Nutrition, College of Natural Sciences, University of Rzeszów, 4 Zelwerowicza Street, 35-601 Rzeszow, Poland; gbartosz@ur.edu.pl

**Keywords:** Tardigrada, dehydration, ionizing radiation, antioxidant, catalase, peroxidase, superoxide dismutase, trehalose, horizontal gene transfer

## Abstract

Tardigrades are unique among animals in their resistance to dehydration, mainly due to anhydrobiosis and tun formation. They are also very resistant to high-energy radiation, low and high temperatures, low and high pressure, and various chemical agents, Interestingly, they are resistant to ionizing radiation both in the hydrated and dehydrated states to a similar extent. They are able to survive in the cosmic space. Apparently, many mechanisms contribute to the resistance of tardigrades to harmful factors, including the presence of trehalose (though not common to all tardigrades), heat shock proteins, late embryogenesis-abundant proteins, tardigrade-unique proteins, DNA repair proteins, proteins directly protecting DNA (Dsup and TDR1), and efficient antioxidant system. Antioxidant enzymes and small-molecular-weight antioxidants are an important element in the tardigrade resistance. The levels and activities of many antioxidant proteins is elevated by anhydrobiosis and UV radiation; one explanation for their induction during dehydration is provided by the theory of “preparation for oxidative stress”, which occurs during rehydration. Genes coding for some antioxidant proteins are expanded in tardigrades; some genes (especially those coding for catalases) were hypothesized to be of bacterial origin, acquired by horizontal gene transfer. An interesting antioxidant protein found in tardigrades is the new Mn-dependent peroxidase.

## 1. Introduction: Tardigrades, the Heroes

**Figure 1:**
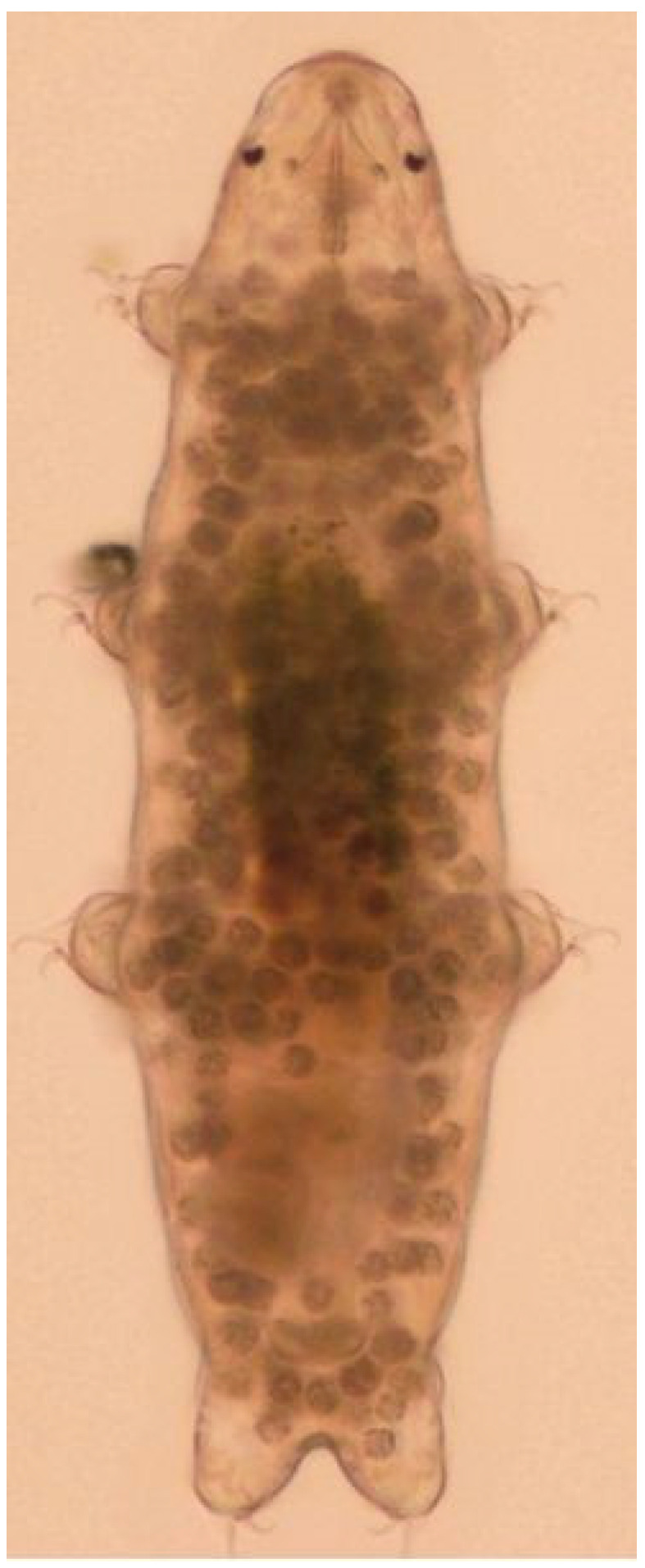
Tardigrade *Hypsibius exemplaris*. Reproduced from [3].

Tardigrades, members of the phylum Tardigrada, commonly called water bears or moss piglets, are microscopic metazoans (50 to 2100 μm, usually 250–500 μm in length), have been fascinating scientists for over 200 years due to the resistance to desiccation and other physical and chemical factors. They were discovered by the German pastor and zoologist J.A.E. Goeze in 1773. Because of their slow movement, the organisms were termed Tardigrada, from Latin *tardigradus*, slow-stepper, by the Italian natural scientist Spallanzani in 1776. They have a bilaterally symmetrical body, composed of five segments; one head segment and four trunk segments, each of the latter with a pair of unsegmented legs ending (most often) in claws [1,2] covered with a cuticle (Figure 1). Their body is covered with a flexible chitinous cuticle, which is smooth or has embedded gibbosities, spines, or plates [2].

Tardigrades show a constant number of cells, even though they are not eutelic. They have a large dorsal brain, often with small eye spots, and a ventral chain of nerve ganglia. A single dorsal sack-like gonad characterizes males, females, and also self-fertilizing hermaphrodites. In several species, there are only females which reproduce by parthenogenesis. Tardigrades are not pathogens for humans [4].

Tardigrades are now classified in the clade Ecdysozoa, among Nematoda, Arthropoda, and five other phyla. Previously, some authors considered tardigrades as closely related to arthropods, while others considered them as members of the Aschelminthes, due to their similarities with nematodes (triradiate pharynx, buccal stylets, and anhydrobiosis). Within Ecdysozoa, Tardigrada belong to the superphylum Panarthropoda and, according to various authors, are thought to be a sister phylum either to Arthropoda, Arthropoda plus Onychophora, or to Onychophora [5].

It has been hypothesized that tardigrades are secondarily miniaturized from a larger ancestor, probably a lobopodian [6,7]. All major organ systems of tardigrades show reductions in the number and size of cells; their five-segmented body might have resulted from the loss of a large middle region [7]. Tardigrades diverged from their closest relatives in the Cambrian more than 500 million years ago. The earliest known true members of the group are known from Cretaceous amber (145 to 66 million years ago) [8,9].

Tardigrades are divided into two major evolutionary lineages represented by Eutardigrada and the more diverse Heterotardigrada mainly on the basis of the structure of the claws, dorsal and cephalic cuticles, body appendages, and reproductive structures [10,11]. A third class, Mesotardigrada, has also been distinguished and so far is represented by a single species and considered to be nomen dubium [12].

Eutardigrada predominantly inhabit limno-terrestrial habitats and harbor the majority of the currently described species [13]. Eutardigrada are morphologically characterized by the presence of a cloaca, a straight midgut, the presence of Malpighian tubules, two double claws on each leg, and the general absence of the numerous cephalic, body and leg sensory organs known from Heterotardigrada.

Heterotardigrada are morphologically characterized by the midgut having lateral diverticula, the presence of a distinct anus and gonopore, and numerous cephalic sensory organs. Heterotardigrada were traditionally and are currently divided into the predominantly marine Arthrotardigrada and Echiniscoidea with tidal, semiterrestrial, and limnic species. Echiniscoidea almost always appear monophyletic, while Arthrotardigrada have been shown to be paraphyletic based on molecular data [14].

Currently, approximately 1400 species have been reported from various habitats, such as marine, fresh-water, or limno-terrestrial environments, though the real number of the tardigrade species is estimated to be much higher [15,16,17,18]. Tardigrades were found in harsh environments such as Antarctica as well as in urban areas, e.g., in activated sludge in a sewage treatment plant. They often constitute the major component of the fauna of lichens and mosses. All tardigrades require surrounding water to grow and reproduce, but in limno-terrestrial habitats, they are subject to occasional periods of desiccation or freezing. When mosses and lichens lose water, tardigrades desiccate or freeze, entering into physiological states called anhydrobiosis and cryobiosis, respectively. Some limno-terrestrial species are able to tolerate almost complete dehydration [19,20,21].

The most important features of tardigrades are summarized in Table 1.

There are two general strategies to cope with water deficiency: the desiccation-avoidance strategy and the desiccation-tolerance strategy [22,23]. The desiccation-tolerance strategy, used for withstanding the dehydrated state, has been adopted by tardigrades. When the surrounding water evaporates, tolerant tardigrades lose almost all body water and enter a metabolically inactive dehydrated state called anhydrobiosis. In such a state of metabolic inactivity, grouped under the general name of cryptobiosis, metabolic activity slows to a near-undetectable rate, until the return of favorable environmental conditions [19,24]. Cryptobiosis was defined as an ametabolic state of life entered by an organism in response to adverse environmental conditions [25]. Cryptobiosis is the main mechanism enabling tardigrades to survive other extreme physical and chemical conditions, apart from dehydration, such as high temperatures, UV radiation, extreme (both high and low) pressure, high osmotic pressure (osmobiosis), oxygen deprivation (anoxybiosis), starvation, and exposure to ethanol and other organic solvents, hydrogen sulfide, carbon dioxide, or magnesium perchlorate [4,26,27,28,29,30]. The hallmark of most tardigrades undergoing cryptobiosis is their remarkable ability to shift into a shriveled anatomical state known as a tun, which they achieve by contracting their limbs, retracting along their medial axis, and in the process expelling their internal water stores. In the process of tun formation, tardigrades may lose most of their body water to form tun [31,32]. Tun formation reduces the body surface area and cuticular permeability, decreasing water evaporation and thus making further desiccation a very slow process. In the case of *Hypsibius exemplaris*, tuns are about 25% of the size of the hydrated tardigrades [33].

Upon exposure to favorable conditions, tardigrades distend and return to active metabolism. Many bacteria and some eukaryotes and bacteria are also capable of cryptobiosis, but no eukaryotes except tardigrades are able to do so across the entirety of their lifespan, including eggs, juveniles, and adults, or in response to such a broad range of stressors [34]. This latent state can be repeated multiple times. Anhydrobiosis does not seem to have effects on animal longevity/senescence in tardigrades [29,35,36]. Nevertheless, the survival of desiccated animals decreases in proportion to the dehydration rate and the time spent in a desiccated state, leading eventually to animal death [37].

The ability of tardigrades to survive dehydration is astounding as the low water content of their body in the tun state is close to complete desiccation. Humans die after a loss of 14% of their body water, and most organisms die when they lose 50% of their water at the body, organ, tissue, or cell level [38]. A possible quantitative definition of complete desiccation is drying to less than 0.1 g H_2_O g^−1^ dry mass (10% water content) or less [22]. The threshold of 10% water content may correspond to the point at which there is not enough water to form a monolayer around macromolecules, which will then lead to the arrest of biochemical reactions and metabolism [39].

Tardigrades have a fairly low lifespan (when continuously hydrated), varying from species to species, averaging at about a few months, but in anhydrobiosis, tardigrades can survive being in this state for about a decade [19,40,41] or longer [21]. A case of revival of a tardigrade tun after 120 years was reported [42], but apparently the longest well-documented survival in the tun state concerns retrieval of living individuals of the Antarctic tardigrade *Acutuncus antarcticus* from frozen phytobenthos after 5 and even after 30.5 years [21]. However, the time to recover may increase with increasing time spent in the dehydrated form [37].

Even in their hydrated active state, tardigrades show an unexpectedly high survival rate upon exposure to adverse conditions that would quickly kill most other known forms of life. Diverse protective mechanisms that remain largely unelucidated helped them to survive fluctuating environments since they evolved in the Cambrian era, and these mechanisms make them probably the most resilient group of multicellular organisms on Earth.

In cryptobiosis, tardigrades are able to withstand extreme chemical and physical conditions; for this reason, they have been called “the toughest animals on the earth” [33]. In most (but not all) cases, the ability of tardigrades to withstand extreme conditions is much higher in the tun form.

Already in the 19th century, tuns of the tardigrade *Macrobiotus hufelandi* were reported to survive a few minutes at 120–125 °C [43]. Rahm found that the tardigrades *Milnesium tardigradum* and *Ramazzottius oberhaeuseri* could survive at 110–151 °C for up to 35 min [44]. More recently, no increase in mortality of *Richtersius coronifer* was found after a 60 min exposure of dehydrated tardigrades to temperatures up to about 70 °C, but at higher temperatures the survival of tardigrades decreased steeply with an LD_50_ temperature of approximately 76 °C, and no animals survived exposure to 100 °C [26]. *Ramazzottius varieornatus* was much more resistant in the dehydrated state to low and high temperatures. No animal survived 1 h exposure at 90 °C in the hydrated state, while about 90% survived it in the dehydrated state [45]. Most (≥76%) of the dehydrated *R. varieornatus* survived 1 h exposure to temperatures of up to 82 °C, but survival dropped to zero at temperatures ≥ 85 °C [18]. In another study, more than 90% of adult anhydrobiotic specimens of *R. varieornatus* survived 1 h exposure to 90 °C [46], and approximately 15% of egg anhydrobiotes survived 80 °C for 1 h [47].

*Richterius coronifer* was found to survive cooling to −196 °C in the hydrated state, showing decreasing viability at a rapid cooling rate (approximately 1500 °C min^−1^) [26]. Most tardigrade species can tolerate cooling rates of 0.31 °C min^−1^, while the most resistant species (*R. oberhaeuseri* and *Paramacrobiotus areolatus*) can tolerate higher cooling rates of 1.38 °C min^−1^ [48]. The survival of dehydrated tardigrades in liquid air for up to 20 months and survival of *R. oberhaeuseri*, *M. tardigradum*, and *Macrobiotus* sp. exposed to −253 °C in their hydrated was reported [49]. Hydrated Antarctic tardigrades (*Echiniscujse nninysi*, *Macrobiotus furciger*, and *Diphasconc hilenense*) showed 24–45% survival after storage at −22 °C for 587 days, while desiccated animals of these species survived in 15–51% storage at −22 °C for 3040 days [50]. Tardigrades were able to survive at a temperature of −272.8 °C, close to absolute zero [48,51].

Desiccated *R. coronifer* is very resistant to alcohols of low polarity although not to alcohols of high polarity. In the dehydrated state, the tardigrade was found to survive in 1-hexanol for 7 days; in 1-butanol, its viability was decreased to 42% after 7 days, while animals exposed to 96% ethanol died within hours [26]. In the case of 1 h exposure of *R. varieornatus* to acetonitrile, survival was 0% for hydrated and about 100% for dehydrated animals [46]. Moreover, the desiccated tardigrade *R. coronifer* was able to survive a 70 h treatment with methyl bromide gas [52].

Tardigrades are very resistant to both low and high pressure. Adult individuals of *M. tardigradum* and *R. coronifer* were able to tolerate a 10-day exposure to vacuum in an open-space environment [53], which strongly suggest that anhydrobiotic tardigrades can survive even in anoxic environments outside Earth. The vast majority (86%) of anhydrobiotic eggs of *R. varieornatus* exposed to low pressure at 5.3 × 10^−4^ Pa to 6.2 × 10^−5^ Pa for 7 days were able to hatch [47].

Desiccated *M. tardigradum* was able to withstand the pressure of 1.2 GPa for 20 min [54] and 7.5 GPa for up to 13 h [55]. The upper lethal limit of a high salt concentration for the tardigrade *R. coronifer* was found to be 500 mOsm/kg [34]. *Hypsibius dujardini* was reported to survive shock pressures at speeds up to of 0.861 GPa [56,57].

These results were the basis for a hypothesis that “tardigrades can travel through the space in a large meteorite’’ [2,58] and prompted experiments devoted to estimate the survival of tardigrades under space flight conditions (Section 3).

## 2. Resistance of Tardigrades to UV and Ionizing Radiation

In particular, tardigrades are resistant to ultraviolet radiation (UV) and show extraordinary resistance to ionizing radiation (IR), with their radiation resistance showing interesting peculiarities.

Tardigrades are capable of tolerating approximately 1000-fold higher dosages of UVB and UVC than human cell lines. A lower tolerance of tardigrades to UV in the hydrated state than in the dehydrated state was reported [58,59], although hydrated tardigrades showed higher tolerance to UV radiation than desiccated ones when irradiated under a low temperature [58]. *Ramazzottius varieornatus* showed a high survival (ca. 80% 5 days post-irradiation) after irradiation with a dose of 2.5 kJ m^−2^ of UVC in the hydrated state, while in the dehydrated state, its survival was around 80% after 20 kJ m^−2^ 13 days after the irradiation, but thereafter, the survival declined. In the dehydrated state, individuals of *R. varieornatus* laid 162 eggs in total after exposure to 2.5 kJ m^−2^ but none after a higher dose of UVC [59].

Studies on cryptobiotic invertebrates, including tardigrades, have shown that tardigrades are also highly tolerant to ionizing radiation [60]. Typically, limno-terrestrial species, e.g., *R. varieornatus*, *M. tardigradum*, *H. dujardini*, and *R. coronifer*, were able to withstand several thousand Gy of radiation (Table 2).

A study of *P. areolatus* showed that this species is very resistant to X radiation, with similar dose responses in the desiccated and hydrated states, and LD_5024h_ values between 5 and 6 kGy [63]. Studies on other γ-irradiated semi-terrestrial or freshwater tardigrade species (*M. tardigradum* [67], *R. coronifer* [65], and *H. dujardini* [62]) brought similar results, with estimates of LD_50_ between 3 and 5 kGy, with doses higher than 1 kGy causing sterility. For comparison, the dose at which 50% of humans die within 30 days is less than 5 Gy [60].

There are two striking features concerning the resistance of tardigrades to ionizing radiation. Firstly, hydrated tardigrades can show similar tolerance to irradiation compared to desiccated animals [61,64,65], which is unexpected given that the indirect effects of IR, contributing significantly to biological effects of radiation, depend on the presence of water. Secondly, the resistance of adult tardigrades to low- and high-LET radiation is similar, and in some cases, even a higher resistance to high-LET radiation was reported [64,67,68]. *M. tardigradum* irradiated with high doses of helium ions (high-LET radiation) tended to survive better in the hydrated state compared to desiccated animals [64]. The biological effect of high-LET radiation involves a lower share of the indirect effect [69]. Both these peculiarities suggest the existence of efficient DNA protection and/or repair pathways [70].

*Hypsibius dujardini* is a freshwater tardigrade with lower tolerance to desiccation compared to limno-terrestrial tardigrades but with a high radiation tolerance in adults, similar to limno-terrestrial tardigrades; it apparently challenges the idea that desiccation and radiation tolerance rely on the same molecular mechanisms [62]. However, tolerance to γ-radiation is clearly lower in the marine tardigrade *Echiniscoides sigismundi*, which is also less tolerant to desiccation, (LD_50_ of about 1.5 kGy seven days post-irradiation) [61].

Radiation resistance is dependent on the life stage of tardigrades. Fertility and developing embryos are generally much more sensitive than adults, and embryos are particularly sensitive in the early stage of development [60,67]. Studies in three tardigrade species (*R. coronifer*, *M. tardigradum*, and *H. exemplaris*) found a similar pattern of radiation sensitivity with respect to the developmental stage, with the early stage being very sensitive, while in the late stage, there was no effect of radiation up to 500 Gy [62,71,72]. Eggs of *R. coronifer* were most radiosensitive in the earliest stage of development, with the LD_50_ dose estimated to be as low as 48 Gy [67]. It has been suggested that the higher radiation tolerance in adults and late-stage embryos of tardigrades compared to early-stage embryos may partly be due to limited mitotic activity, since tardigrades have a low degree of somatic cell division and dividing cells are known to be more sensitive to radiation [62]. Fertility is more radiosensitive than the viability of tardigrades, apparently for the same reason. *H. exemplaris* can survive and reproduce after exposure to 100 Gy. After exposure to 500 or 2000 Gy (about half of the LD_50_), individuals of this species survived well but no longer reproduced [60,62].

Apart from the efficient DNA protection and repair, a well-developed antioxidant defense system has been suggested as a possible explanation for the high radiation tolerance seen among tardigrades [60,62] (see Section 5).

## 3. Tardigrades in Space

The extraordinary resistance of tardigrades to extreme conditions, including low pressure and high-energy radiation was the stimulus for studies of their behavior under space flight conditions.

A few space programs included tardigrades at a low Earth orbit. In 2007, three projects were conducted during the FOTON-M3 mission studies. The Tardigrade Resistance to Space Effects (TARSE) Project was the first one involved in the mission of FOTON-M3. Its aim was to analyze the impact of environmental stress, life history traits, and DNA damage in space (on board the spacecraft) on the eutardigrade *P. richtersi*. In that project, active and anhydrobiotic tardigrades were exposed to radiation in microgravity conditions. During the flight, active tardigrades molted and laid eggs which developed into normal animals that were able to reproduce. Both active and inactive individuals had high survival rates with no induction of heat shock proteins (HSPs) (70 and 90 kDa) and showed no formation of DNA double-strand breaks. After the flight, both desiccated and hydrated animals showed high survival rates, induction of the antioxidant response, and no induction of HSP expression [73,74].

The next project involved in the mission of FOTON-M3 was TARDIS (Tardigrada In Space). The main goal of this project was to check if tardigrades from two species, *M. tardigradum* and *R. coronifer*, were able to survive conditions of open space. Desiccated tardigrades were exposed in space during 10 days at a low Earth orbit (258–281 km above sea level) to the combined effect of space vacuum (10^−6^ Pa), cosmic radiation (100 mGy), and UV radiation of two different spectra (UVA + B: 280–400 nm, total dose 7095 kJm^−2^; UV_vacuum_ to UVA (116.5–400 nm), total dose 7577 kJ m^−2^) at a low Earth orbit. Compared to samples protected from solar radiation, which were not affected significantly by the exposure to cosmic radiation and space vacuum, UV radiation-exposed tardigrades suffered a high mortality and no animals of the two species survived the full UV spectrum. However, 12% of the *M. tardigradum* specimens exposed to UVA + B survived, thus being the first animals ever shown to survive the combined exposure of vacuum, cosmic radiation, and UV radiation under space conditions (so far, only bacteria and lichens were reported to have survived the combined exposure to space vacuum and solar/galactic cosmic radiation) [53]. No eggs hatched when exposed to these factors simultaneously, while exposure to space vacuum plus cosmic radiation had no significant effect on hatchability [75]. In *R. varieornatus* irradiated with UVC (254 nm), thymidine dimer formation was much lower in the desiccated than in the hydrated state [59]. The experiments showed that tardigrades can survive exposure to the space vacuum, but the addition of factors such as UV solar radiation, ionizing solar radiation, and galactic cosmic radiation significantly reduced their survival rate [53,75].

In the third project from the FOTON-M3 mission, RoTaRad (Rotifers, Tardigrades, and Radiation), the effects on immediate survival, long-term survival, and fecundity of the selected species of limno-terrestrial tardigrades in extreme stress conditions (mainly cosmic radiation) were studied [76].

The next project was TARDIKISS (Tardigrades in Space). In this project, two tardigrade species, *P. richtersi* and *R. oberhaeuseri*, in the desiccated (anhydrobiotic) state stayed for 16 days on board the Space Shuttle Endeavour (its last mission STS-134) docked to the International Space Station. The main aim of this project was to broaden the knowledge of life history traits and mechanisms of DNA repair during exposure to space flight stresses. The first results confirmed that microgravity and cosmic radiation did not significantly affect the survival rate of tardigrades [77].

The ability to enter into cryptobiosis is helpful for travelling for long cosmic distances, but also for providing a possibility of surviving long periods when environmental conditions are extremely unfavorable. This could enable researchers to determine whether tardigrades can survive and live on other planets in the solar system or on their moons [2]. Tardigrades, in particular *P. experimentalis*, were suggested to be suitable for future astrobiological studies concerning Mars, as it shown that this species has an ability to demonstrate long-term resistance to magnesium perchlorate at levels highly exceeding those observed naturally on Earth and being in the range that can be expected on Mars (up to 1.5 mM) [27].

The survival ability of the tardigrade *R. varieornatus* was examined in the anhydrobiotic state at a burial depth of 5 mm in regolith after a 40-day exposure to simulated Martian environments with temperatures ranging from −40.4 °C to 24.0 °C, 19.3 Wm^–2^ of UV flux (200–400 nm), 10–22 mbar of atmospheric pressure, and 95.3% CO_2_. Over 70% percent of the tardigrades survived after exposure to the simulated Martian environments, implying that tardigrade anhydrobiotes and other anhydrobiotic multicellular organisms can survive on Mars [78].

## 4. What Makes Tardigrades So Tough?

There are good reasons to suspect that the resilience of tardigrades to various adverse conditions has its evolutionary and mechanistic roots in the resistance to desiccation. Therefore, an understanding of the resistance to dehydration seems crucial to explain the resistance of tardigrades to most adverse conditions.

**Figure 2:**
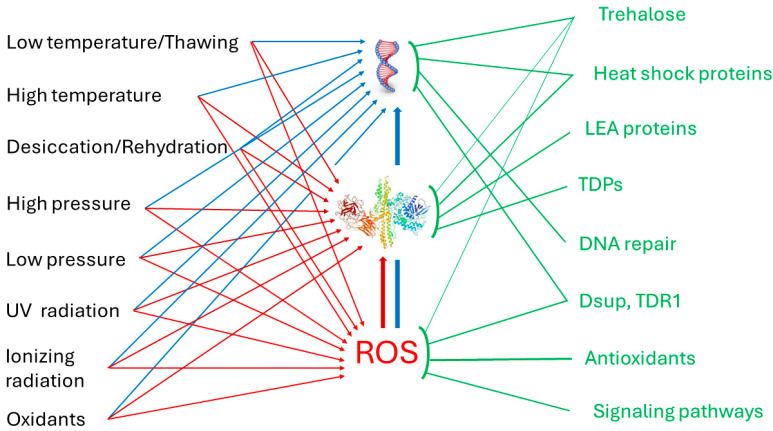
Scheme of the mechanism of action of various environmental factors and main protective mechanisms in tardigrades. All these factors inflict damage on DNA and proteins and oxidative stress (increased generation of reactive oxygen species (ROS)). ROS themselves can damage DNA and proteins. Protective mechanisms are shown in green. TDPs, tardigrade disordered proteins; Dsup, damage suppressor protein.

Dehydration is known to cause severe damage to organisms at the membrane level as well as to their proteins [79]. The survival strategy, during early stages of dehydration, seems to consist in avoiding protein unfolding and membrane disturbances. Tolerance to radiation in tardigrades seems to represent mainly (though not totally, taking into account their resistance to IR in the hydrated state) a cross-tolerance of anhydrobiosis [60], and the overlapping pathway is presumably the defense against reactive oxygen species (ROS) that mediates protein oxidation and efficient DNA damage. The main targets of action of various physical and chemical factors on tardigrades, and the main mechanisms of defense against them are summarized in Figure 2.

### 4.1. Trehalose

Some organisms entering anhydrobiosis synthesize different kinds of molecules working as bioprotectants. Small-molecular-weight molecules can work during the dehydration phase, stabilizing proteins and membranes. Trehalose (α-D-glucopyranosyl-(1→1)-α-D-glucopyranoside) is the main compound of this type. This non-reducing disaccharide has been shown to accumulate to very high concentrations in various anhydrobiotic animals, including nematodes, brine shrimp embryos, and sleeping chironomid larvae [11]. It is known to be a membrane stabilizer [80] and is thought to form a glassy state under dry conditions [81]. However, in contrast to some other invertebrate groups with anhydrobiosis where trehalose levels in the dry state reach 15–20% of the dry weight (nematodes, *Artemia*, and the sleeping chironomid *Polypedilum vanderplanki*), tardigrades have much lower levels of trehalose (below 3%), and only in one family (Macrobiotidae) is its level upregulated in response to desiccation [82,83], while many tardigrade species produce low or undetectable levels of the disaccharide [49,82,83,84,85]. Some tardigrades, like *H. exemplaris*, appear to entirely lack the trehalose 6-phosphate synthase and trehalose 6-phosphatase enzymes required for trehalose synthesis [10,82,86].

### 4.2. Heat Shock Proteins

Heat shock proteins (HSPs), acting as molecular chaperones, are widely distributed in tardigrade cells, and many are upregulated during tardigrade anhydrobiosis, which suggests their essential role in cryptobiosis [87,88,89]. Apart from 70 kDa and 90 kDa HSPs, nine small heat shock proteins (sHSPs) were identified in the tardigrade *H. exemplaris*, with many of them highly expressed. These proteins may promote desiccation tolerance by limiting desiccation-induced protein aggregation. Two sHSPs of this species, HSP21 and HSP24.6, limited desiccation-induced aggregation and loss of function of a model enzyme, citrate synthase [90,91].

### 4.3. Late Embryogenesis-Abundant (LEA) Proteins

The term “molecular shield” was first proposed to describe the function of LEA proteins that may provide a protective shell that shields proteins during desiccation and prevent aggregation [92,93]. The proposed function of molecular shield proteins is to limit the number and frequency of intermolecular interactions that could lead to protein aggregation during desiccation, but their mode of action is different from that of molecular chaperones. LEA proteins may also act as hydration buffers, membrane stabilizers, and ion sinks [94,95,96].

### 4.4. Tardigrade-Unique Proteins

Studies in eutardigrade omics showed the presence of so-called “tardigrade-unique” proteins that are cytoplasmic-, secreted-, and mitochondrial-abundant heat-soluble (CAHS, SAHS, and MAHS, respectively) and possess sequences without conservation in other phyla, collectively known as tardigrade disordered proteins (TDPs), that have been suggested to play critical roles in cellular protection upon anhydrobiosis and other stresses [10,87,96,97,98,99,100]. These proteins have a unique cold-shock domain, which gave rise to a hypothesis that the cold-shock protein acts as an RNA chaperone involved in the regulation of translation following freezing. The non-specific binding of tardigrade-specific proteins to RNA prevents the formation of secondary structures, thus keeping the mRNA in a linear form essential for translation [15]. However, the “tardigrade-unique proteins” seem to be missing in the heterotardigrade lineage, revealing that cryptobiosis in general cannot be generally attributed to these proteins.

### 4.5. DNA Repair Proteins

It was concluded on the basis of a lack of differences in the sensitivity to IR between hydrated and dehydrated tardigrades that radiation tolerance in tardigrades is not due to biochemical protectants connected with the desiccated state. Rather, cryptobiotic tardigrades may rely on efficient mechanisms of DNA repair [65].

Tardigrades were shown to save all major pathways of DNA repair (non-homologous end joining, homologous recombination, mismatch repair, nucleotide excision repair, and base excision repair). An exception seems to be the marine heterotardigrade *E. sigismundi*, which was reported to lack pathways for non-homologous end joining (c-NHEJ), one of the major pathways for repair of DNA double-strand breaks in addition to homologous recombination [15,101]. This species is tolerant to rapid desiccation [101], but seems to be less tolerant to long-term anhydrobiosis compared to semi-terrestrial tardigrades [102] and to IR [61].

In *H. exemplaris* exposed to IR or bleomycin, genes involved in base excision and single-strand repair were the most highly induced, with the responses to both agents overlapping to a high extent [103,104].

### 4.6. Damage Suppressor Protein (Dsup)

During sequencing of the genome of the desiccation- and radiation-tolerant tardigrade *R. varieornatus*, Hashimoto et al. discovered a tardigrade-unique protein, damage suppressor (Dsup), that strongly associates with nuclear DNA and is able to preserve chromosomal DNA from hydroxyl radical-mediated cleavage [1,105]. Dsup is a nucleosome-binding protein enriched (to more than 60%) with serine, alanine, glycine, and lysine (SAGK) residues, which are disordering amino acid residues. Dsup protein is highly basic (pI = 10.55), especially in the C-terminal region, suggesting its potential association with DNA through electrostatic interactions. The C-terminal region of the protein is necessary and sufficient for its association with DNA [105]. As hydroxyl radicals react primarily with hydrogen atoms exposed in the minor groove of DNA, the protection of DNA by Dsup suggests that Dsup predominantly blocks access to the minor groove [106].

Transfecting human embryonic kidney cells (HEK293) with the gene coding for Dsup) resulted in improved viability and a reduction in DNA damage by up to 40% after irradiation with X-rays, compared to irradiated non-transfected cells [105]. The reduced DNA fragmentation in Dsup-expressing cells was likely due to the reduced occurrence of DNA breaks rather than the facilitation of the DNA repair process. This notion was further evidenced by the fact that Dsup-expressing cells exhibited a much lower number of the DNA break marker γ-H2AX, which accumulates shortly after irradiation and is usually retained for several hours, even after completion of DNA break repair [1] and were more resistant to H_2_O_2_ treatment, which only marginally involves the pathways responsible for DNA repair. However, the expression of Dsup also protected HEK cells against UV radiation. After UV treatment, DNA is not physically protected by Dsup after UV-C irradiation, but rather Dsup activates more efficient mechanisms of damage repair (STAT1, c-Myc, p-c-Jun; ATR, and BCRA1), XRCC6 protein, which participates in the UV-G2 checkpoint and ERCC6, whose gene represents a potential target for inactivation by UV light and seems to act as a “dosimeter” of DNA damage [107]. When expressed in plants, Dsup affected the expression of the endogenous genes involved in DNA damage signaling and repair [108]. It is suggested that, in addition to a direct interaction and coverage of DNA, Dsup induces protection by altering the expression levels of endogenous genes critical for cell survival and proliferation. This conclusion is in line with a recent finding, contrasting with previous studies that in *H. exemplaris*, the rate of single-strand breaks induced was roughly equivalent to that in human cells, suggesting that DNA repair plays a predominant role in the remarkable resistance of tardigrades to IR [103].

However, Dsup has only been found in two species within the Hypsibioidea superfamily [103], so, apparently, these proteins were acquired after the divergence of the class Eutardigrada (as they are not present in the other class, Heterotardigrada) [15,109]; thus, the necessary and sufficient set of genes and pathways enabling anhydrobiosis and resistance to other extreme conditions still remain elusive.

### 4.7. TDR1 Protein

TDR1, a protein broadly conserved in tardigrades, is among the most highly expressed genes upon IR and bleomycin treatment. Based on its high proportion of basic amino acid residues, the TDR1 protein can likely bind tightly to DNA at a rate of one TDR1 protein every three bases and appears to form large DNA protein aggregates and can directly shield DNA from ROS. TDR1, like DUSP1, strongly interacts with DNA in vitro. While DUSP1 and TDR1 do not share sequence similarity, both contain many basic residues. The expression of both DUSP1 and TDR1 in mammalian cells leads to a reduced burden of DNA damage [103,110].

### 4.8. Stress-Signaling Pathways

Tardigrades lack highly conserved networks for stress regulation, including those that connect hypoxia, genotoxic stress, and oxidative stress to the conserved master regulator target of rapamycin (TOR) [105]. However, the ATP-dependent differential phosphorylation of the AMP-activated protein kinase (AMPK) regulatory network following anhydrobiosis revealed that protein phosphatase 2A activation is essential to successfully induce tuns via anhydrobiosis [111,112,113]. In *R. varieornatus*, eight genes were found to be lost in the highly conserved stress-responsive signaling pathways. Three of these genes, HIF1A, PHD, and VHL, are central components to regulate the response to hypoxia. REDD1 is a downstream target of HIF1A25, as well as a downstream target induced by p53 on genotoxic stress. REDD1 activates the TSC1/TSC2 complex, leading to downregulation of the mammalian target of rapamycin complex 1 (mTORC1) activity. The other lost gene, coding for sestrin, is also a downstream gene of p53 connecting genotoxic stress to mTOR signaling. As TSC1/TSC2 is activated by oxidative stress, the tardigrade lacks the signaling components connecting various stresses, such as hypoxia, genotoxic stress, and oxidative stress, to downregulate mTORC1. It was hypothesized that the tardigrades avoid excessive destruction of cellular components after severe stress by suppressing autophagy induction and it may be beneficial to resume cellular activity by using partially damaged biomolecules after rehydration. In this respect, tardigrades are less sensitive to environmental stress [105]. In *H. exemplaris*, AMP-activated protein kinase (AMPK) was activated during the preconditioning stage for anhydrobiosis and inhibition of its activity impaired successful anhydrobiosis [112].

### 4.9. Other Mechanisms

Many other mechanisms may contribute to the resistance of tardigrades. The role of the antioxidant defense is discussed below. Tardigrades rely on mitochondrial activity for desiccation survival; using chemical uncouplers of the mitochondrial electron transport chain prevents tun formation after exposure to anhydrobiotic conditions [114]. The flux of calcium in and out of the mitochondria is likely implicated in tun formation, and the voltage-gated anion channel 2 (VDAC2) closes to decrease flux in response to external stressors [33].

### 4.10. Horizontal Gene Transfer

Part of the defensive system of tardigrades may be due to horizontal gene transfer, which was suggested to occur especially efficiently in this group of animals. A study of *H. dujardini* demonstrated that 17.5% of tardigrade genes were gained by horizontal gene transfer (HGT). For instance, glutathione synthase in tardigrades appears to be derived from bacteria and this species appears to have replaced catalase genes with catalase genes acquired from foreign sources. Desiccation-tolerant organisms might be especially susceptible to taking up and incorporating foreign DNA into their genomes from their environment rather than exclusively from endosymbionts. When desiccated membranes are rehydrated, they become transiently leaky, making the uptake of large macromolecules possible; this phenomenon has been exploited to introduce large nucleic acids and drugs into the cytoplasm of rehydrating anhydrobiotic cells [115]. However, another research group offered a counterargument, suggesting that a substantial portion of the genes recognized as acquired by horizontal gene transfer was derived from contaminating microorganisms [116]. In *R. varieornatus*, the proportion of putative HGT genes was estimated to be only 1.8% at most. Extensive horizontal gene transfer is thus not a common feature in the phylum Tardigrada and is also not correlated with the tolerance of extreme conditions, because *R. varieornatus* has superior tolerability compared with *H. dujardini* [105].

## 5. Role of Oxidative Stress and Antioxidants

### 5.1. Oxidative Stress Accompanies Environmental Stress

A common feature of environmental stress, such as heat, cold, dehydration, osmotic shock, and UV-radiation, is the generation of ROS and change in cellular redox potential. Oxidative stress seems to be one of the most deleterious effects of water depletion. For example, after dehydrating the cells of yeast *Saccharomyces cerevisiae*, ROS generation is 10-fold higher than that of hydrated cells [117]. Desiccated cyanobacteria show increased ROS levels, especially in the presence of light [118]. Also in plants, enhanced formation of ROS occurs during drought and chilling [119]. In the moss *Fontinalis antipyretica*, the production of ROS was found to be associated with the dehydration rate. Low levels of ROS were detected in leaves dehydrated very slowly, whereas significantly higher values were found in leaves that were rapidly dehydrated [120].

It has been hypothesized that animals which suppress metabolism in response to stress have the advantage of a slow metabolic recovery, decreasing the production of ROS and thereby limiting cellular and genomic damage [121]. A complete metabolic shutdown, preventing ROS generation from internal sources, coupled with apparent species-specific adaptations within antioxidant defense systems could explain how tardigrades seemingly diminish the deleterious effects of ROS damage and thereby ensure post-cryptobiotic survival [62]. However, even in anhydrobiosis, the generation of ROS takes place. Their origin is not yet well known; they may be produced both during the dehydration (entrance phase) and desiccated states. Dehydration reduces the hydration shell of biomolecules, slows down cytoplasmic and intracellular transport, and, by causing shrinkage of cells, concentrates all cellular molecules, increasing ionic strength and altering the pH of the cytoplasm. These changes might produce dysfunctions in specific enzymes or promote chemical reactions that would not normally occur in a fully hydrated cell. In the desiccated state, ROS production most probably occurs both through nonenzymatic reactions and auto-oxidations rather than through enzymatic reactions. Nevertheless, the possibility that enzymatic reactions occur during the desiccated state cannot be completely excluded even if they proceed at very low rates [122,123]. Moreover, during the rehydration phase, as the hydration level of the tissue increases and the glass transition temperature approaches, the trapped free radicals become unstable and are released. Free radical release and free radical content measured by EPR increase significantly after the vitreous state is melted [79,123,124].

The production of superoxide was demonstrated by spin trapping in the tardigrade *P. richtersi* not subjected to any stressor. Spin trapping showed an increased intracellular level of superoxide following the induction of osmotic stress [125]. In *P. spatialis* storage cells, the level of ROS estimated with dihydrodichlorofluorescein diacetate (H_2_DCF-DA) increased during the desiccation state and increased further upon rehydration [126].

The enhanced production of ROS, such as superoxide, hydrogen peroxide, and the highly reactive hydroxyl radical, leads to a deregulation of redox-sensitive pathways as well as oxidative modification of essential biomolecules (e.g., DNA, proteins, and lipids). Lipid peroxidation produces a decrease in the fluidity of biological membranes and their fusion, which interferes with membrane permeability during rehydration [79]. In desiccated specimens of *P. richtersi*, the level of lipid peroxidation products reacting with thiobarbituric acid was more than 5 times higher than in hydrated animals [127]. An accumulation of protein carbonyls during the anhydrobiotic period was reported in tardigrades. Irradiation with UVC was also shown to increase the level of protein carbonylation in *H. exemplaris* [128]. It was reported that *M. tardigradum* accumulates DNA damage during storage in the anhydrobiotic state and this damage reaches a high level after 10 months of an anhydrobiotic period, although little or no damage was observed during short periods of anhydrobiosis [129]. Furthermore, the process of desiccation itself induces no or only minor DNA damage [29].

### 5.2. Oxidative Stress Can Be Necessary for Cryptobiotic Survival

Tardigrades survive multiple stressors that are prone to generating ROS in vivo, including desiccation, extreme fluctuation in osmolarity, and freezing [49,130,131]. Surprisingly, oxidative stress seems necessary for tun formation and tolerance of extreme conditions by tardigrades, perhaps as a signal inducing the metabolic response.

*H. exemplaris*, like other tardigrades, is shown to respond to exogenously applied ROS by forming tuns in a dose-dependent manner. Hydrogen peroxide (0.75–5 mM) induced tun formation that was inhibited when cysteine thiols were irreversibly blocked. Rapid exposure to reducing conditions or a reduction in oxidized thiol groups with β-mercaptoethanol caused tun release and death. EPR spectroscopy demonstrated an intracellular release of ROS following the stressor action. The inhibition of voltage-dependent anion channel protein 2 generates tuns, and ROS control of this ion transporter is likely implicated in tardigrade stress. ROS induction of tun formation through the oxidation of cysteine thiols was examined by blocking cysteine thiols in hydrated tardigrades with either iodoacetamide or N-ethylmaleimide, both of which irreversibly bind to reduced cysteine thiols and make the formation of disulfide bonds impossible. Both these compounds prevented the H_2_O_2_-induced tun formation. Antioxidant pretreatment (with glutathione, ergothioneine, and β-lapachone) prior to osmotic stress decreases tardigrade survival. These results indicate that intracellularly generated ROS mediate tardigrade survival in the presence of exogenous stressors by inducing cysteine oxidation, which is necessary for cryptobiotic survival, even when tun formation is not involved [33].

### 5.3. Antioxidant Defense of Tardigrades

**Figure 3:**
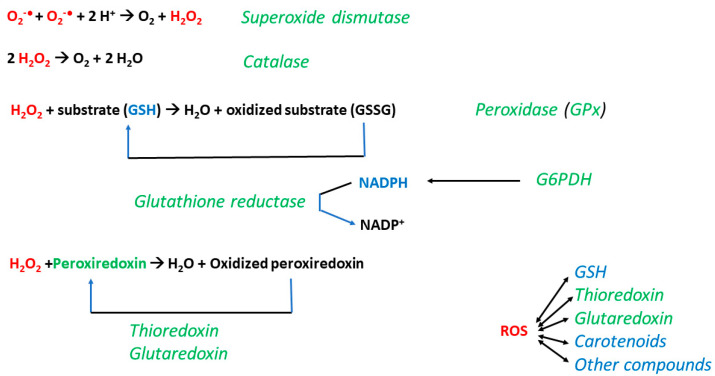
Basic antioxidant system of tardigrades. GSH, glutathione; GSSG, oxidized glutathione; GPx, glutathione peroxidase; ROS, reactive oxygen species. Red, ROS; green, antioxidant proteins; blue, small-molecular-weight antioxidants. Superoxide dismutases (SODs) disproportionate superoxide radicals to oxygen and hydrogen peroxide. Catalases disproportionate hydrogen peroxide to water and molecular oxygen. Peroxidases reduce hydrogen peroxide at the expense of oxidation of other substrates; glutathione peroxidases (GPxs) oxidize glutathione. Peroxiredoxins reduce hydrogen peroxide, and other ROS are reactivated by thioredoxins. Glutathione disulfide reductase regenerates the reduced form of glutathione at the expense of NADPH, generated mainly by glucose-6-phosphate dehydrogenase. Thioredoxins as well as glutaredoxins are small (12 to 14 kDa) proteins which may also directly react with ROS and reactivate other proteins oxidized by ROS. Enzymes of glutathione biosynthesis (γ-glutamylcysteine ligase and glutathione synthetase) are important for maintaining appropriate level of glutathione, the main intracellular antioxidant, or elevating this level, if needed. Glutathione-*S*-transferases (GSTs) conjugate reactive electrophiles to glutathione [132].

Tardigrades share the antioxidant system common to the whole animal world (Figure 3), with some interesting peculiarities.

The tripeptide glutathione (γ-Glu-Cys-Gly) is the main antioxidant in the majority of eukaryotic cells. It reacts directly with free radicals and other ROS, and with electrophiles. Glutathione peroxidases (GPxs) reduce hydrogen peroxide oxidizing glutathione to glutathione disulfide (GSSG), which can be reduced by glutathione reductase (GR) at the expense of NADPH, produced mainly by glucose-6-phosphate dehydrogenase, the first enzyme of the pentose phosphate cycle. Glutathione *S*-transferases (GSTs) catalyze conjugations of exogenous and endogenous electrophiles (including electrophilic lipid peroxidation products) to glutathione (GSH), which results in their detoxification.

Usually, the first ROS formed in biological systems is superoxide radical anion (O_2_^−•^). Superoxide dismutases (SODs) dismutate superoxide to molecular oxygen and hydrogen peroxide. The latter is disproportionated to molecular oxygen and water by catalases, or reduced to water by GPxs. Hydrogen peroxide is also scavenged by peroxiredoxins that become oxidized in the process of its reduction. Oxidized peroxiredoxins are reduced by thioredoxin reductases, again at the expense of NADPH.

Antioxidant defenses may prevent damage to tardigrade proteomes and protect DNA repair enzymes under hostile conditions. This enables the effective repair of DNA and the elimination of severely damaged molecules to recover cellular integrity.

Some metabolic sources of ROS seem to be eliminated in this group of animals, alleviating metabolic oxidative stress. In peroxisomal β-oxidation, acyl-CoA oxidases catalyse the initial conversion of acyl-CoA and produce hydrogen peroxide as a side product. Loss of peroxisomal oxidative enzymes including those in β-oxidation was noted in the tardigrades that probably leads to decreased hydrogen peroxide production during fatty acid metabolism. On the other hand, in mitochondria, a similar conversion is catalyzed by acyl-CoA dehydrogenases, which produce FADH_2_ instead of hydrogen peroxide. Alternative oxidase (AOX)-coding genes were also identified in the genomes of tardigrade species [10,15]. Though primarily associated with plants, there is increasing evidence that AOX can be found across some species from all eukaryotic kingdoms; one of the physiological functions of this enzyme is the limitation of ROS production in mitochondria [133].

Tardigrade species have a considerable number of gene-encoding proteins involved in antioxidant defense [15] (Table 3).

A comparison of the of tardigrade genomes with other metazoans revealed a characteristic expansion of several stress-related gene families including those coding for SODs. Sixteen SODs, likely located within mitochondria, cytosol, and also peroxisomes, were found in *R. varieornatus*, and it seems to be a general tardigrade feature, with eutardigrades possibly having a second round of duplication (12–16 putative genes) as compared to heterotardigrades (7 putative genes in *E. sigismundi*). In *M. tardigradum*, both CuZn superoxide dismutases (SODs) (six contigs) and MnSODs (two contigs) have been identified. CuZn-SODs are highly expressed in *R. varieornatus*, while a somewhat lower expression is seen in *E. sigismundi* [134,135].

For comparison, less than ten SODs are found in most metazoans, and three genes coding for various SOD forms are present in humans [15,105,109].

Model structures of several *R. varieornatus* (Rv) SODs were investigated, and it was found that some of them are also unusual enzymes of this group, with features such as deletion of the electrostatic loop or β3 sheet and unusual metal-binding residues. In RvSOD15 CuZnSOD, histidine in position 87 is substituted with valine. These studies show that RvSOD15 and some other RvSODs may have evolved to lose the SOD function, suggesting that gene duplications of antioxidant proteins do not solely explain the high stress tolerance of anhydrobiotic tardigrades [136].

Genomic, transcriptomic, and proteomic studies showed that the catalase gene family seems expanded within Eutardigrada (four copies of the catalase gene), while the picture of the catalase gene family is more complex in Heterotardigrada. No transcripts of catalase were found in the transcriptome of the marine heterotardigrade *E*. *sigismundi*. In *R. varieornatus*, three catalases and one putative pseudo-gene were found. The putative decrease in hydrogen peroxide production is consistent with the loss of typical metazoan catalases (clade III) in the tardigrade genome [105]. On the other hand, reciprocal blast searches against available EST data of the limno-terrestrial heterotardigrade *E. testudo* revealed eight catalase sequences, indicating a complex picture for the evolution of the catalase gene family within tardigrades [15]. Catalases are classified into three sub-groups, termed clades I, II, and III, and all other metazoan catalases are classified as clade III. Interestingly, as suggested for *R. varieornatus* and *H. exemplaris*, multiple sequence alignments and Pfam domain searches revealed that *R. coronifer*, *H. dujardini*, and *R. varieornatus* possess a catalase structure that resembles the bacterial clade II, containing an additional PF01965 domain, common to bacterial catalases. It supports the suggestion that parachelan eutardigrades likely obtained their catalase genes through HGT [10,105]. Bacterial clade II catalases exhibit greater resistance to denaturing conditions, such as high temperature or 7 M urea than metazoan clade III catalases, and, thus, tardigrade clade II catalases might be active even in hyperosmotic conditions during dehydration/rehydration and contribute to desiccation tolerance.

The superfamily of glutathione transferases (GSTs) builds a further cellular detoxification system. All tardigrade species seem to have a large number of soluble GSTs. In *M. tardigradum*, 27 different contigs were found that belong to the GST superfamily. Soluble glutathione *S*-transferases (GSTs) are the most highly expressed genes in *E. sigismundi* and *R. varieornatus*, whereas in *R. coronifer*, CuZn-SODs exhibit the highest expression followed by GSTs. GSTs are induced upon a variety of stressors; they are characterized by wide substrate specificity, high activity, and inducibility in response to various toxicants. The ubiquitous occurrence of GSTs among tardigrades indicates that enzymes of this group play a fundamental role in tardigrade antioxidant defense [135].

Animal peroxidase genes were far more abundant in the genomes of invertebrates than vertebrates and substantially expanded in tardigrades [137]. Glutathione peroxidase was reported to be crucial for successful anhydrobiosis in *P. spatialis* [126,138].

The identification of *R. varieornatus* genes responsible for the cross-tolerance of anhydrobiosis and UVC led to the discovery of the g12777 gene coding for a novel Mn^2+^-dependent peroxidase. The expression of this gene in HEK293 cells augmented the resistance of these cells to oxidative stress induced by hydrogen peroxide. The protein product of this gene localizes mainly in the Golgi apparatus, binds Zn^2+^ and Mn^2+^, and shows peroxidase activity only in the presence of Mn^2+^ ions, reducing H_2_O_2_ with specific activity equivalent to 5% of the specific activity of bovine catalase. Unlike most Mn peroxidases, this protein does not rely on heme for the peroxidase function. This protein, also called anhydrobiosis-related Mn-dependent peroxidase (AMNP) and conserved throughout the phylum Tardigrada, has a disulfide bond at the active site and seems to represent a new class of peroxidases [139].

In the eutardigrade *M. tardigradum*, different isoforms of peroxiredoxins (eight contigs) and 14 thioredoxin domains were identified [134,135,140], although no studies about their regulation during desiccation have been conducted.

A comprehensive comparative analysis of the proteome of tardigrades in three different states: early embryonic state (EES) and adult tardigrades in the active and anhydrobiotic (tun) states showed that GSTs are approximately 3-fold higher in adult *M. tardigradum* compared to the EES, which is probably due to the exposure to higher amounts of endobiotics and xenobiotics. Eggs are laid inside the old cuticle and remain there during embryonic development. Therefore, embryos are not directly attacked by xenobiotics. In contrast, CuZn SODs are upregulated in the EES compared to adults. This suggests that the protection of embryos from oxidative stress is a prerequisite for their development and plays an important role in development. A comparison of active and tun states of *M. tardigradum* showed upregulation of GSTs and peroxiredoxins in the active state and, in contrast, upregulation of SODs in the tun state [138]. In *P. richtersi*, the content of GSH; total antioxidant capacity; amounts of catalase, glutathione peroxidase, and thioredoxin; and activities of GPx and GR increased, but the activity of catalase decreased [124,127].

Tardigrades are endowed with a standard set of small-molecular antioxidants, headed by GSH. Peculiarities in this respect include the presence of carotenoids and a possible contribution of trehalose to the antioxidant defense in some tardigrade species.

Non-photosynthesizing organisms are incapable of synthesizing carotenoids de novo, and animals must obtain them through their diet and can convert them into new derivatives. Some tardigrades, especially of the genus *Echiniscus*, contain carotenoid pigments. The exact structure of these carotenoids has not been characterized. They are located in the body cavity, but not in the cuticular structures; remarkable interindividual differences in total carotenoid content were observed. The carotenoids present in tardigrades might well have a photoprotective function. The observation that most pigmented tardigrade species dwell in sun-exposed habitats, such as glacier surfaces [141] or on mosses and lichens growing on rocks located at high altitudes and latitudes supports this hypothesis.

The carotenoids present in the heterotardigrade *Echiniscus blumi* are derived from food but are about 20 times more concentrated than in the moss *Grimmia orbicularis*, which is the food of the tardigrades. A decrease in the amount of carotenoids (mainly β-carotene) after the induction of oxidative stress by H_2_O_2_ in *E. blumi* suggests that carotenoids in tardigrades work as scavengers for ROS formed, i.e., during exposure to dehydration [124,142].

Trehalose is a non-reducing sugar, so it is hard to expect that it can be a direct-acting antioxidant. However, trehalose was proposed to have an antioxidant role, in addition to being a cryoprotectant, although the data supporting this view come from in vitro studies or experiments on other organisms. In yeast cells, trehalose was shown to attenuate oxidant-induced alterations of proteins induced by exposure to H_2_O_2_ [143]. This sugar was capable of reducing the intracellular ROS level in yeast cells during dehydration [117]. In vitro, trehalose significantly reduced the oxidation of unsaturated fatty acids through a weak interaction with the double bonds [144].

### 5.4. Induction of Antioxidant Defense by Environmental Factors

Tardigrades have been shown to upregulate antioxidant defense mechanisms during cryptobiosis and the actions of other harmful environmental conditions [127,145,146]. One explanation for this phenomenon is provided by the theory of “preparation for oxidative stress” [147]. Organisms adapted to stressors such as freezing, dehydration, salinity variations, hypoxia, or anoxia often reduce their metabolic rates to maximize their chances of survival. However, upon recovery of environmental conditions and basal metabolic rates, cells are affected by an oxidative burst. This process results in a large production of ROS, which, if uncontrolled, can cause extensive damage to cellular components, potentially leading to irreparable cell damage and death [148]. Thus, a number of adapted organisms are able to increase their antioxidant defenses during the action of the stressor [147].

Several studies based on genomics, transcriptomics, proteomics, as well as biochemical assays have documented the expression of a wide variety of known antioxidants in desiccated tardigrades compared to hydrated ones [15,127,135,137,141].

In *P. richtersi*, increased SOD and glutathione peroxidase activities and glutathione levels were reported in response to dehydration, suggesting its importance in the process [130]. Moreover, the upregulation of catalase-encoding genes during anhydrobiosis was detected in the tardigrade *H. exemplaris* [10]. In the same species, upregulation of GSTs was reported in response to desiccation [10,112,134].

In total, the level of ROS estimated by the oxidation of H_2_DCF-DA in *P. spatialis* after 3 and 12 h rehydration increased with the duration of desiccation until day 20 [126].

In desiccated specimens of the tardigrade *P. richtersi*, the total amount of glutathione (expressed in the number of nmoles/animal) was significantly (more than 3-fold) higher than that measured in hydrated animals, but the activities of catalase and glutathione reductase was not significantly higher in desiccated tardigrades compared to hydrated ones. The activities of GPx, glutathione reductase, and SOD (expressed in mU/animal) were higher in the desiccated state; the GPx was the most abundant antioxidant enzyme detected, and its amount increased with desiccation [127]. The crucial role played by GPx in anhydrobiosis in *P. spatialis* was confirmed by experiments showing that animals targeted for GPx gene disruption by RNAi are not able to survive after a cycle of desiccation and rehydration. Targeting the GR and catalase genes resulted in the immobility of the tardigrades immediately after rehydration, but then they recovered their motility [126].

A 2.5-fold induction of expression of the g12777 gene, coding for Mn-dependent peroxidase was found during the slow desiccation of *R. varieornatus* [10].

The effects of dehydration and subsequent rehydration were studied in two tardigrade species, *P. spatialis* and *A. antarcticus*. No significant differences in the GSH content and GR and GPx activities were observed. Catalase activity increased in the dehydrated state in *P. spatialis* but showed no changes in *A. antarcticus*, while SOD activity decreased during dehydration in *A. antarcticus* but showed no changes in *P. spatialis* These results were interpreted as proof of the high constitutive expression of some antioxidant enzymes in these species [149]. 

The exposure of *R. varieornatus* to UV led to the induction of 1 314 genes, genes coding for GST, thioredoxin, and peroxiredoxin. Many of these genes were also induced during tardigrade anhydrobiosis [10], suggesting similar pathways are being regulated between desiccation and UVC exposure. Yoshida et al. found 141 genes upregulated under both conditions; among them, genes related to antioxidative stress, coding for GST and SODs [139].

Tardigrades have a high tolerance toward Cu^2+^; one reason for this resistance may be the high content of antioxidant proteins [146].

In the TARSE project, active and dehydrated eutardigrade *M. richtersi* spent 12 days in space as part of the FOTON-M3 mission. In active but starved tardigrades, space flight induced an increase in the GSH content and GPx activity, and no changes in the GR activity (with a decreasing tendency). The activities of catalase and SOD decreased significantly [73].

In the TARDIKISS project, two tardigrade species, *P. richtersi* and *Ramazzottius oberhaeuseri*, in the desiccated (anhydrobiotic) state stayed for 16 days on board a space shuttle, and no significant changes were found in the level of GSH and activities of most antioxidant enzymes [150].

## 6. Discussion: What Can We Learn from Tardigrades?

Tardigrades, among other cryptobiotic animals, have evolved solutions to a number of challenging biological conditions that can be of considerable interest to medicine. Studies of mechanisms underlying anhydrobiosis and radiotolerance are relevant not only for a better understanding of these intriguing natural phenomena, but also for the development of novel technologies for radioprotection and biological preservation. The protective role of trehalose on membrane systems and proteins gave rise to medical applications, including the preservation of liposomes [123] and blood platelets [151,152].

The outstanding resistance of tardigrades to IR is especially interesting since DNA damage by IR is a serious threat especially for cancer patients undergoing radiation therapy and for astronauts and future Moon or planetary missions. A threat of use of nuclear weapons by terrorists or autocrats is continuously present. The mechanisms of protection against ionizing radiation are of particular interest, as it seems that tardigrades have unique protective proteins. The Dsup and TDR1 proteins were postulated to be potential agents that could protect from DNA strand breaks. While potential medical applications of LEA proteins can be foreseen in the future [106], the production of more resistant plants and animals is within reach. Experiments of this type have already been performed. The expression of Dsup in rice increased UV tolerance, increased grain size, altered starch granule structure, and changed the expression of many genes [153]. Transgenic *Drosophila. melanogaster* expressing Dsup was found to be more resistant to ionizing radiation and hydrogen peroxide, but since the protein also acts as a non-specific transcriptional repressor and RNA-binding protein, its expression may lead to a disturbance of a number of biological processes in the fruit fly [154]. Thus, the results and side effects of such experiments should be scrutinized and monitored.

## 7. Conclusions

The amazing resistance Tardigrades have to various physical and chemical factors seems to be mainly a consequence of their resistance to dehydration. Various factors seem to contribute to this resistance, among them, the antioxidant system and its induction during desiccation, proteins protecting DNA against damage, and enzymes of DNA repair. The specific mechanisms conditioning tardigrade resistance to various factors are of considerable interest for biotechnology and medicine.

## Figures and Tables

**Table 1:** Characteristic features of Tardigrada.

Common Names	Water Bears; Moss Piglets
Systematic position	Phylum of Ecdysozoa
Number of species	About 1400
Occurrence	In mosses and lichens
Food	Bacteria, algae, plants, and small invertebrates (species-dependent)
Size	50–2100 µm
Number of cells	The same within species
Body	Barrel-shaped, four pairs of legs, and legs without joints
Body cover	Cuticle containing chitin
Reproduction	Oviparous, some species parthenogenic
Lifespan	3 months–2 years
Resistance	Extreme resistance to various physical and chemical factors
Mechanisms of resistance	Mostly (but not only) cryptobiosis

**Table 2:** Radiation sensitivity of tardigrades. According to [1], modified.

Species	State	Ionizing Radiation	Radiation Sensitivity	Reference
*Echiniscoides sigismundi* *	H * H	γ (6.22 Gy min^−1^) γ (6.22 Gy min^−1^)	1591 Gy (LD_50/24h_) 1449 Gy (LD_50/7d_)	[61]
*Hypsibius dujardini*	H	γ (6.04 Gy min^−1^)	4180 Gy (LD_50/48h_)	[62]
*Hypsibius exemplaris*	H	Cs^137^ (1.4251 Gy min^−1^)	4360 Gy (LD_50_)	[62]
*Paramacrobiotus areolatus*	D *	X	5700 Gy (LD_50/1d_)	[63]
*Milnesium tardigradum*	H D H D	γ (5.5 to 61.7 Gy min^−1^) γ (5.5 to 61.7 Gy min^−1^) ^4^He (50 MeV, 16.3 keV μm^−1^) ^4^He (50 MeV, 16.3 keV μm^−1^)	5000 Gy (LD_50/48h_) 4400 Gy (LD_50/48h_) 6200 Gy (LD_50/48h_) 5200 Gy (LD_50/48h_)	[61,64] [61,64] [61,64] [61,64]
*Ramazzottius varieornatus*	H D	^4^He (50 MeV) ^4^He (50 MeV)	4000 Gy ~100% survival 4000 Gy ~90% survival	[46] [46]
*Richtersius coronifer*	H H D D D D	γ γ γ Protons (2.55 MeV) X ^4^He, ^56^Fe	4700 Gy (LD_50/18h_) 2500 Gy (LD_50/30d_) 3000 Gy (LD_50/22h_) 10,240 Gy (LD_50/24h_) 2000 Gy 2000 Gy	[65] [65] [65] [65] [66] [66]
*Hypsibius exemplaris*	H	Cs^137^ (1.4251 Gy min^−1^)	4360 Gy (LD_50_)	[62]

H, hydrated; D, dehydrated; * marine species.

**Table 3:** Number of genes coding for antioxidant enzymes in chosen Tardigrada species (adapted from [15], modified).

Enzyme	Tardigrade Species				Humans
	*Echiniscoides sigismundi*	*Richtersius coronifer*	*Ramazzottius varieornatus*	*Hypsibius exemplaris*	*Homo* *sapiens*
	**Number of genes coding for the enzyme**				
Superoxide dismutase	8	14	17	15	3
Catalase	0	4	4	4	1
Peroxiredoxins	5	7	9	12	6
Thioredoxins	12	13	10	12	14
Glutaredoxin	5	4	3	3	4
Glutathione disulfide reductase	1	1	1	1	1
Glutathione peroxidase	2	1	1	1	8
Glutathione synthetase	1	2	2	3	1
Soluble glutathione *S*-transferases	35	30	31	34	22
Microsomal glutathione *S*-transferases	0	2	0	2	4
Glucose-6-phosphate dehydrogenase	1	1	1	2	1

## Data Availability

No new data were created.
